# Management of Hydrocele

**Published:** 1875-05

**Authors:** 


Management of Hydrocele.—Mr. E. Lund, of the Manchester Hospital, in a recent report, says "the secret of success in the management of hydrocele consists in thoroughly distending the sac of the hydrocele, by first noting the number of fluid ounces of serum drawn off, and then injecting at least one ounce more of port wine, so as thoroughly to expand and fill the sac."

A gentleman saw an advertisement that a receipt for the cure of dyspepsia might be had by sending two postage stamps to the advertiser, and the answer was: "Dig your own garden, and let whisky alone."

The duck is the Egyptian symbol for a physician.

A NOVEL has been recently issued in England, the hero of which is an enthusiastic homœopathist; and by its practice he is enabled to cure the most hopeless cases, to win fame and wealth, and at last to marry his sweetheart, who at one time rejected him because he embraced the doctrines of homœopathy. The wicked persons of the "tale" believe in contrary medical theories, and are continually shown up in very damaging positions throughout the whole volume. This is certainly a *novel* way of propagating medical theories, and has been already opposed by some of the adherents of homœopathy, who argue that a scientific discovery is not best advanced by placing it on a level with fairy tales.—*Pharmacal Gazette.*

